# Targeted Next Generation Sequencing (NGS) to Diagnose Hereditary Hemolytic Anemias

**DOI:** 10.18502/ijhoscr.v14i3.3726

**Published:** 2020-07-01

**Authors:** Rishab Bharadwaj, Thulasi Raman, Ravikumar Thangadorai, Deenadayalan Munirathnam

**Affiliations:** 1 Department of Paediatric Hematology/Oncology, Kanchi Kamakoti CHILDS Trust Hospital, Chennai, India; 2 Department of Pathology, Kanchi Kamakoti CHILDS Trust Hospital, Chennai, India; 3 Department of Pediatrics, Kanchi Kamakoti CHILDS Trust Hospital, Chennai, India; 4 Department of Paediatric Hematology/Oncology, Kanchi Kamakoti CHILDS Trust Hospital, Chennai, India

**Keywords:** Hereditary hemolytic anemia, Dehydrated hereditary Stomatocytosis, Kӧln hemoglobinopathy, Targeted next generation sequencing

## Abstract

Hereditary hemolytic anemias present a unique diagnostic challenge due to their wide phenotypic and genotypic spectrum. Accurate diagnosis is essential to ensure appropriate treatment. We report two cases, which presented as hemolytic anemias, but initial workup was inconclusive and they were finally diagnosed with the help of Next Generation Sequencing (Dehydrated Hereditary Stomatocytosis and Kӧln Hemoglobinopathy). The introduction of gene sequencing to aid diagnosis of these disorders is a revolutionary step forward and should be incorporated earlier in the workup of such patients.

## Introduction

 Hereditary hemolytic anemias are a clinically heterogeneous disease entity which comprise of red blood cell (RBC) membrane disorders, hemoglobin disorders, and RBC enzyme disorders. Due to wide phenotypic and genotypic spectrum, they present a unique diagnostic challenge. We report two such rare cases which were confirmed with the help of gene sequencing. 

## Case presentation

**Case 1**

**Figure 1:**
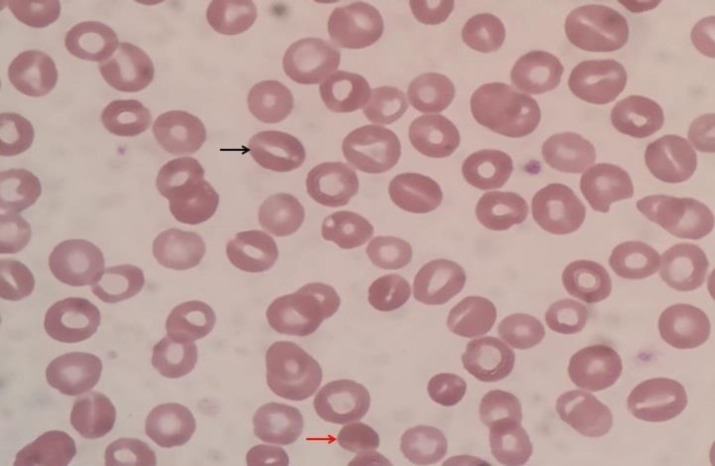
x1000 magnification. Photomicrograph showing few stomatocytes and occasional spherocytes

A second born child of third degree consanguineous marriage presented to us at the age of 25 days of life with pallor. There was no significant antenatal history. Birth weight was 2700 grams. The child had been admitted elsewhere at 12 hours of life with pallor and jaundice. Hemoglobin was 5.8 g/dL, with total bilirubin of 12.8 mg/dL. Reticulocyte count was 3.5%. Both baby and mother blood group was O positive. The child was transfused and advised to follow up. At the time of presenting to us, Hemoglobin was 5 g/dL and Reticulocyte count 3%. Peripheral smear showed few microspherocytes with normocytic, normochromic anemia. Direct Coomb's test was negative. Hemoglobin electrophoresis was normal. Minor blood group incompatibility was negative. The child was investigated for TORCH infections and Parvovirus B19 which was negative. The child received packed red cells transfusion and was followed up. 3 weeks later, Hemoglobin had again dropped to 5.2 g/dL. A bone marrow aspiration was done at 3 months of age which showed marked erythroid hyperplasia, with normal myeloid cell line and megakaryocytes. The child received periodic blood transfusions, once in 3-4 weeks. At the age of 6 months, peripheral smear showed few microspherocytes, some stomatocytes and evidence of hemolysis (Figure 1). 

Osmotic Fragility testing revealed shift of curve to the left. A clinical exome assay for hereditary hemolytic anemia revealed a heterozygous missense mutation in exon 10 of the *PIEZO1 *gene (chr16:88804004G>C; Depth: 126x) resulting in the amino acid substitution of Glutamine for Histidine at codon 386 (p.His386Gln; ENST00000301015.9)**,** which has been reported to cause Dehydrated Hereditary Stomatocytosis (DHS). 

**Case 2**

**Figure 2:**
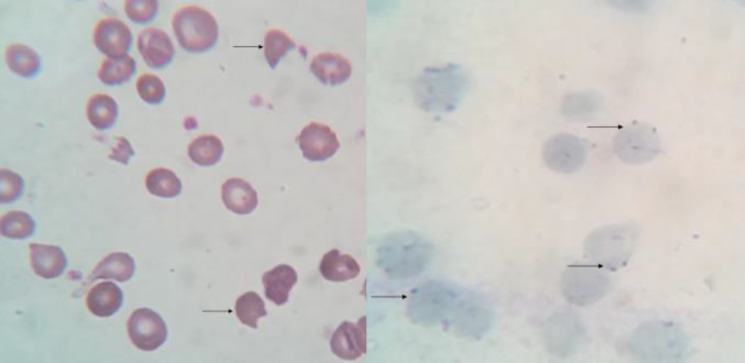
On the left - x1000 magnification – Leishmann stain. Arrows showing bite cells.

A 7-year old, previously well child presented with complaints of passing high-colored urine. On examination, he was pale and icteric. Hemoglobin was 7.1 g/dL, Reticulocyte count 10%. Peripheral smear showed few microspherocytes with evidence of hemolysis (Figure 2). 

On the right - x1000 magnification – Supravital stain. Arrows showing membrane-bound Heinz bodies

Direct Coomb's test was negative. G6PD level was normal. Osmotic fragility test was negative. Hemoglobin electrophoresis revealed HbA 90.3%, HbA2 3.2%, HbF 1.7% and an unknown Hemoglobin variant (4.8%). He was started on oral Prednisolone at a dose of 2mg/kg/day. Repeat Hemoglobin showed 10.5 g/dL and steroid was gradually tapered. On follow-up, although Hemoglobin was relatively stable, he had persistent reticulocytosis. Clinical exome assay for Coomb’s negative hemolytic anemia was done which revealed a heterozygous missense variation in exon 2 of the HBB gene (chr11:5247827C>T; Depth: 133x) that results in the amino acid substitution of Methionine for Valine at codon 99. This has been reported to cause a rare unstable hemoglobin variant, Hb Kӧln.

**Details about NGS panel used** 

DNA extracted from blood was used to perform targeted gene capture using a custom capture kit. The sequences obtained were aligned to human reference genome (GRCh37/hg19) using BWA program and analysed using Picard and GATK version 3.6 to identify variants relevant to the clinical indication. Clinically, relevant mutations were annotated using published variants in literature and a set of disease databases - ClinVar, OMIM, GWAS, HGMD and SwissVar. Common variants were filtered based on allele frequency in 1000Genome Phase 3, ExAC, EVS, dbSNP147, 1000 Japanese Genome and internal Indian population database. Only non-synonymous and splice site variants found in the clinical exome panel consisting of 8332 genes were used for clinical interpretation.

## Discussion

 No relevant, significant family history was found in immediate family, and no details could be obtained about extended family in either of the cases. This could be explained by ignorance regarding the medical condition as the patients were from the lower socio-economic class. Hereditary stomatocytoses (HSt) constitute a wide spectrum of hemolytic disorders in which the erythrocyte membrane cation permeability is increased, with reduced intracellular potassium and increased sodium content resulting in deregulation of cellular volume ^1^. This causes morphological abnormality of RBCs with the presence of stomatocytes on the peripheral blood smear. Despite the highly variable clinical presentation of HSt, almost all the forms present with hemolysis and anemia, which can vary from mild to severe. Gain-of-function mutations in PIEZO, encoding a mechanosensitive cation channel, and deleterious mutations of KCNN4, encoding the Gardos channel have been implicated^2^. Andolfo et al.^1^ noted that 29 families with PIEZO1 mutations have been reported to date. The PIEZO1 mutations described until now in DHS1a-b patients are several. Most of them consist of missense modifications. Although the majority of the mutations are private, three recurrent variants (Leu2495_Glu2496dup, Arg2456His, and Thr2127Met) accounted for more than 50% of all mutated alleles in DHS1a-b patients^3^ and PIEZO1-related patients showed mild hemolytic anemia, not improved post-splenectomy, and most developed severe thrombosis. A variable percentage of stomatocytes can be found (<20% in DHS and >20% in Overhydrated Hereditary Stomatocytosis (OHS)). Osmotic gradient ektacytometry is a useful tool to diagnose these conditions. It shows a leftward shift of the minimum in the deformability index (Omin) at low osmolarities. However, it is not available in all the diagnostic centres. Occasionally, transfusions are needed for intermittent hemolysis. Despite the low rate of transfusions, HSt are considered to be iron loading anemias and iron chelation plays a key role in management. As recently described in the recommendations of the European Haematology Association, splenectomy is contraindicated in both DHS and OHS because of the increased risk of thromboembolic complications, and also because it is ineffective in DHS. The role of bone marrow transplantation has not been conclusively established. 

Unstable hemoglobins are a group of genetic variants of hemoglobins in which the mutation of amino acids into alpha and beta globins affect the structure of the molecule, making it unstable. Some hemoglobins are discreetly unstable and are not associated with clinical symptoms, while other unstable hemoglobins precipitate with great intensity, causing hemolytic anemia, excretion of “free heme” such as dipyrroles, making the patient’s urine dark ^4^. Kӧln haemoglobin was first described by Pribilla et al in 1965, where they found a “new” haemoglobin in haemoglobin electrophoresis, forming a small band between HbA and HbA2, constituting around 5% of total haemoglobin. Since then, although cases have been reported in Europe and North America, to the best of our knowledge, this is the first such report in a child from India. Patients with Hb Kӧln usually present a mild hemolytic anemia characterized by reticulocytosis, splenomegaly, and elevated circulating bilirubin and lactate dehydrogenase (LDH) levels. They rarely require transfusions and are managed conservatively. Although splenectomy has been reported to be effective^5^, most reports have not found it to be beneficial. 

Hereditary haemolytic anemias pose a diagnostic challenge, with many patients undergoing repeated, unsuccessful investigations over many years. This is mainly due to the great phenotypic variability observed in these disorders. Accurate genetic diagnosis of these conditions is important not only for achieving a definitive diagnosis but also for determining appropriate genetic counselling, prognosis, and treatment. Panel-based genetic tests offer a lot of advantages over the traditional specialized investigations used to diagnose hereditary anemias ^6^. Targeted NGS is a powerful tool which should be incorporated earlier in the work-up of such patients.
